# Uncommon orbital metastasis in ductal breast carcinoma: a rare presentation 12 years after treatment

**DOI:** 10.1093/jscr/rjae428

**Published:** 2024-06-27

**Authors:** Kenza Horache, Manal Jidal, Kenza Sidki, Youssef Omor, Rachida Latib, Sanae Amalik

**Affiliations:** Radiology Department, National Institute of Oncology, University of Medicine and Pharmacy of Rabat, Av. Allal Al Fassi, Rabat, Morocco; Radiology Department, National Institute of Oncology, University of Medicine and Pharmacy of Rabat, Av. Allal Al Fassi, Rabat, Morocco; Radiology Department, National Institute of Oncology, University of Medicine and Pharmacy of Rabat, Av. Allal Al Fassi, Rabat, Morocco; Radiology Department, National Institute of Oncology, University of Medicine and Pharmacy of Rabat, Av. Allal Al Fassi, Rabat, Morocco; Radiology Department, National Institute of Oncology, University of Medicine and Pharmacy of Rabat, Av. Allal Al Fassi, Rabat, Morocco; Radiology Department, National Institute of Oncology, University of Medicine and Pharmacy of Rabat, Av. Allal Al Fassi, Rabat, Morocco

**Keywords:** orbital metastasis, ductal breast carcinoma, enophtalmosis, long-term surveillance

## Abstract

Orbital metastasis originating from breast carcinoma, particularly ductal carcinoma, represents a rare clinical entity, with lobular carcinoma usually being more common. Long-term surveillance in breast cancer patients is crucial for early detection of metastasis. Herein, we present a case of a 70-year-old woman with a history of left ductal breast carcinoma, diagnosed and treated 12 years ago. She then developed left eye vision loss, diplopia, enophthalmos, and chemosis in October 2024. Imaging revealed orbital metastasis involving the left superior and lateral rectus extraocular muscles. Biopsy confirmed the diagnosis of orbital metastases arising from ductal breast carcinoma. This case underscores the significance of long-term surveillance in breast cancer patients, as metastasis can manifest years after the initial diagnosis. Despite its rarity, orbital metastasis warrants consideration in the differential diagnosis of ocular symptoms in patients with a history of breast carcinoma. Treatment primarily aims at palliation and preserving visual function, with prognosis typically poor.

## Introduction

Orbital metastasis from breast carcinoma represents a rare but clinically significant phenomenon, typically arising from lobular carcinoma, with ductal carcinoma constituting a minority of cases. Herein, we present a case of orbital metastasis in a 70-year-old woman with a history of left ductal breast carcinoma, diagnosed and treated 12 years prior. The case highlights the unusual occurrence of orbital metastasis from ductal breast carcinoma and emphasizes the importance of long-term surveillance in breast cancer patients for early detection and management of metastatic disease.

## Case presentation

A 70-year-old woman, previously diagnosed with left ductal breast carcinoma 12 years ago, negative for HER2 and progesterone receptors but positive for estrogen receptors (ERs), staged as pT2N1. She underwent a mastectomy and lymph node removal, followed by chemotherapy and 5 years of tamoxifen hormone therapy. She then continued with follow-up examinations. No significant abnormalities were noted until October 2024 when she was referred to our oncology institute due to vision loss in her left eye, accompanied by diplopia, enophthalmos, and chemosis.

**Figure 1:**
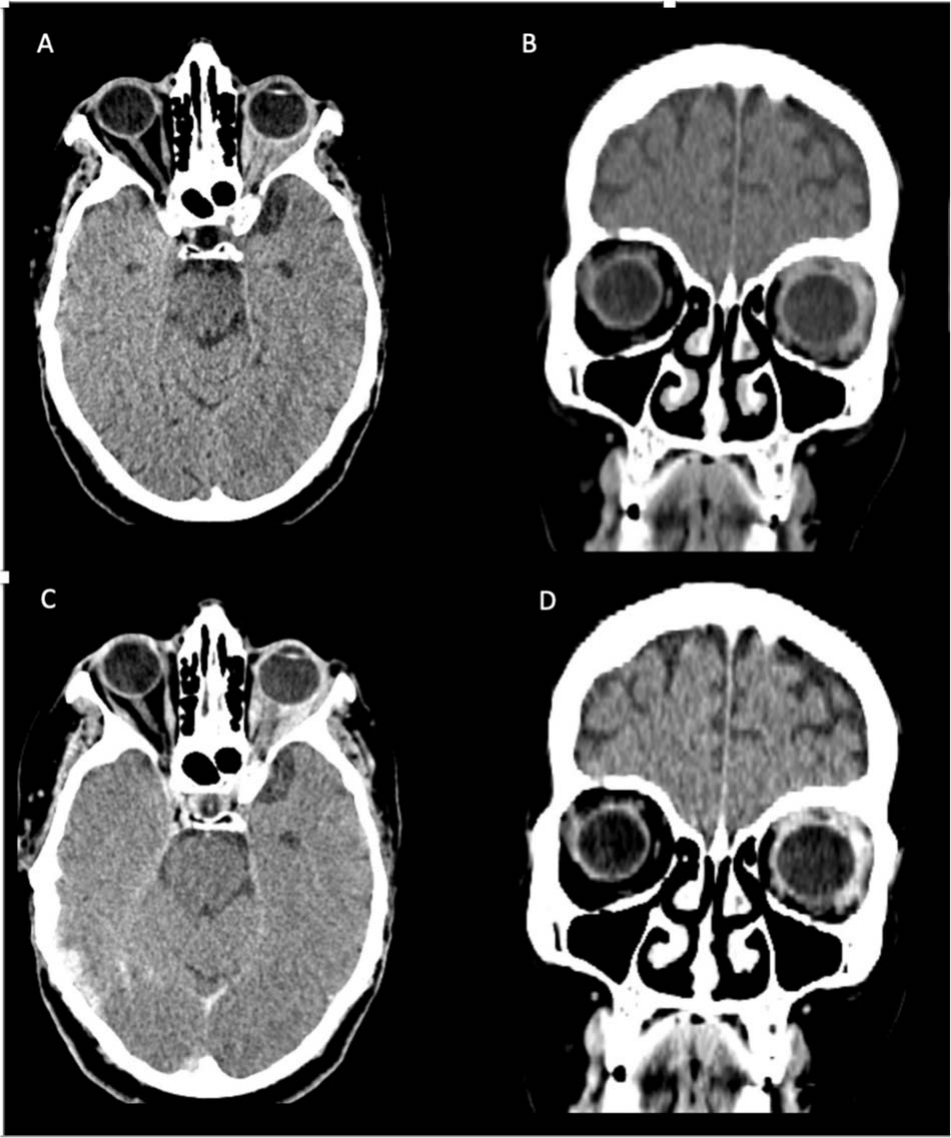
Axial and coronal cerebral CT scans show enlargement of the left lateral and superior rectal muscles (A–B), with slight enhancement (C–D).

The CT scan displayed a tissue infiltration and bulking of the left superior and lateral extraocular rectus muscle (Fig. 1).

**Figure 2:**
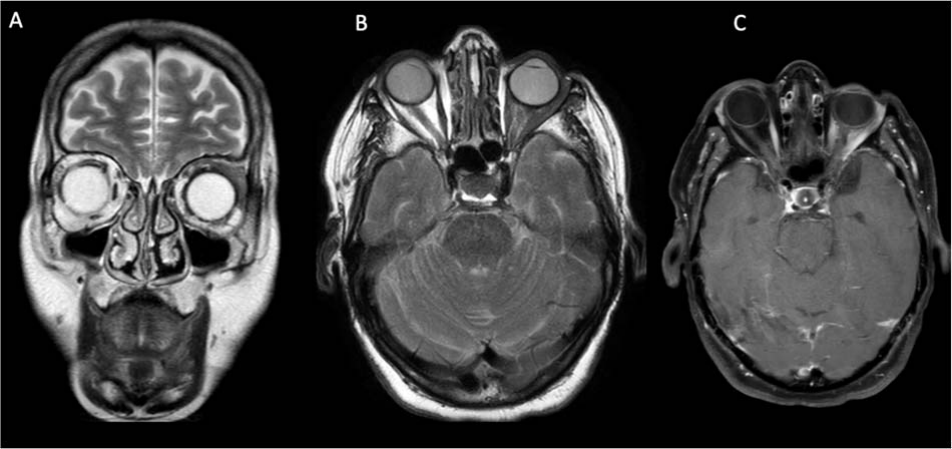
Cerebral and orbital MRI. (A–B) Coronal and axial T2-weighted MRI show iso-intense thickening of the left lateral and superior rectal muscles with fat atrophy. (C) Axial T1-weighted MRI with gadolinium enhancement and fat suppression reveals slight enhancement of the left lateral and superior rectal muscles with infiltration of the surrounding tissues.

MRI scan showed a lump involving the left superior and lateral extraocular rectus muscles, with slight enhancement along with infiltration of surrounding fat tissue between the ophthalmic nerve and lateral rectus muscle (Fig. 2).

**Figure 3:**
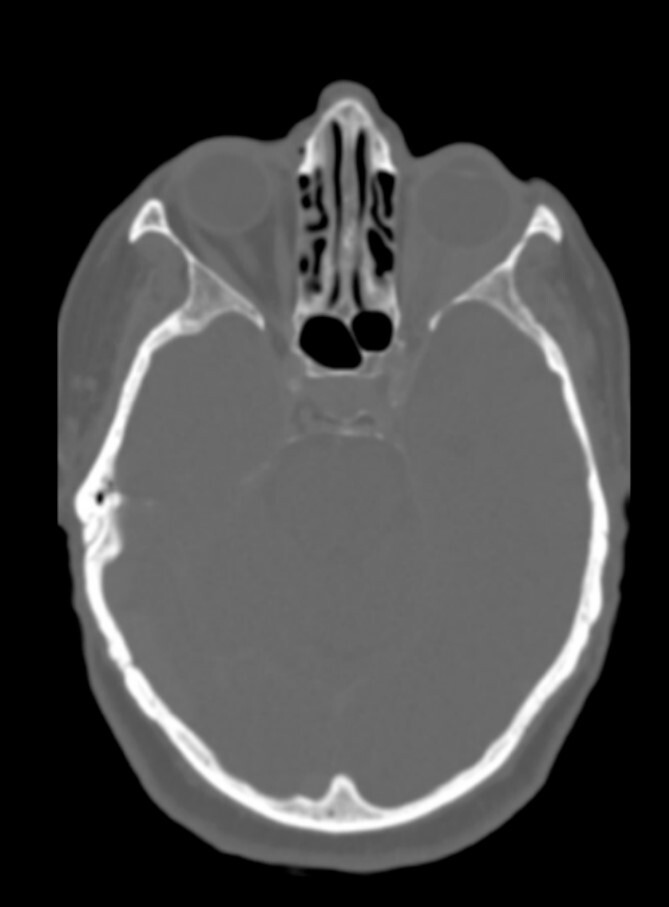
No cranial bone lesions were detected.

There was no evidence of involvement of the central nervous system, and no cranial bone lesions were detected in the CT scan (Fig. 3).

**Figure 4:**
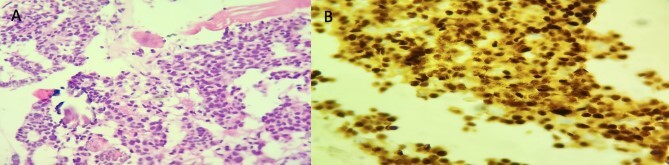
Histopathologic examination of left orbital biopsy specimen. (A) HE stain (×200) tumor cells infiltrating striated muscle fibers. (B) Immunohistochemistry: ER (×200) diffuse and strong nuclear staining.

The patient underwent transpalpebral biopsy, revealing tumoral infiltrative growth within the striated muscle fibers and immunohistochemistry is showing a diffuse and strong expression of ERs (Fig. 4).

These findings were consistent with orbital metastasis originating from nonspecific breast ductal carcinoma.

**Figure 5:**
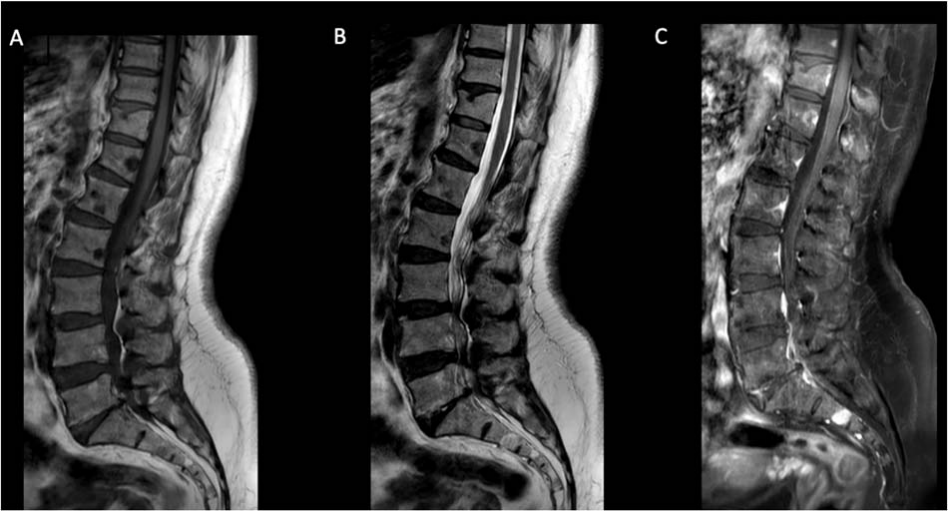
Spine MRI. Sagittal T1 (A) and T2 (B) reveal multiple hypo-intense vertebral metastases with enhancement (C).

Further imaging tests showed multiple vertebral metastases but no organ dissemination (Fig. 5).

The patient is currently undergoing orbital radiotherapy combined with chemotherapy and hormone therapy as well as zolendronic acid. No significant improvement was noted.

## Discussion

This case exhibits two unusual characteristics:

1. The rare occurrence of orbital metastasis arising from ductal breast carcinoma, considering that orbital metastases predominantly originate from lobular carcinoma.
2. The metastatic tumor manifested 12 years following the initial breast carcinoma.

Metastasis to the eye can be categorized into two types: ocular and orbital metastases [1].

Generally, orbital metastasis occurs less frequently compared to ocular metastasis, accounting for 3% of all orbital lesions and 10% of orbital tumors [1, 2].

It locates in orbital fat tissue and extraocular muscles, from where it may invade the orbital bone [3].

Bilateral orbital metastases are less frequent than unilateral ones, usually caused by breast carcinoma (20% compared to 4% from other sources) [4].

The most common sources of orbital metastases are represented by the breast (49–53%), followed by the prostate gland (12%), lung (8%), and melanoma [4].

Raap *et al.* reviewed a series of 14 orbital metastases. The orbital metastases were derived from breast cancer in 8/14 cases, 7 of which were classified as metastatic lobular breast cancer. Other entities included non-small cell lung cancer (4/14), infiltrating ductal breast cancer (1/14), prostate cancer (1/14), and adenocarcinoma of the esophagus (1/14) [5].

Among the histologic types of breast cancer, the frequency of ductal carcinoma is as high as 78%, whereas the frequency of lobular carcinoma is ~11% [6]. Despite this, orbital metastasis originating from lobular carcinoma are predominant, occurring five times more frequently [5], and only a few cases of orbital metastasis from ductal breast carcinoma, as was the case for our patient, has been reported. [3] The progression of lobular carcinoma is propelled by estrogen stimulation, elucidating the notable prevalence of positive ERs observed in biopsy specimens [4]. However, the histological characteristics and immunohistochemical (IHC) features of orbital metastases may occasionally vary from the primary tumor [7].

In patients with breast carcinoma, the time interval between the diagnosis of breast cancer and the development of eye metastasis varies greatly, ranging from 1 month to 25 years [8].

Freedman *et al.* analyzed the charts of 112 patients and revealed that it took ~4 years from the initial breast cancer diagnosis to the occurrence of metastasis to the eye and orbit [9]. Nevertheless, in some cases, this interval can be longer, as demonstrated in the case being discussed. Age is also a significant factor, with orbital metastases more prevalent in individuals aged 60 or older [4].

In the case of orbital metastasis, the initial symptom typically manifests as diplopia, with pain and exophthalmos caused by the mass effect [1, 10]. Additionally, cases of enophthalmos, as was the case for our patient, have been documented, particularly in metastases originating from scirrhous breast carcinoma and gastric cancer [1].

In non-contrast CT scans, metastatic lesions in the orbit usually present as irregularly shaped masses that have similar density to muscle tissue. These masses may exhibit slight enhancement [11].

MRI is considered the best imaging modality for diagnosis [8]. It can reveal two distinct patterns of orbital metastases: one characterized by a mass appearance and exophthalmos, and the other one by an infiltrating appearance [12].

While imaging modalities are valuable for diagnosis, a conclusive determination is made through histological analysis of the lesion. Two techniques are commonly employed: fine needle aspiration or open surgical biopsy. In cases with strong clinical history and suggestive imaging features, this biopsy step may be omitted [12]. However, it should be strongly considered prior to treatment planning [6].

The treatment of orbital metastasis is usually palliative. It primarily aims to manage the growth of the metastatic mass, preserve visual function, and enhance the patient’s quality of life [4].

Orbital surgery is primarily diagnostic but may also serve palliative purposes, such as palliative decompression, orbitotomy, and tarsorrhaphy [12]. However, extensive orbital surgery to remove the metastasis is not recommended due to the high risk of complications.

External beam radiation therapy is the best modality to control tumor size and maintain visual function. In some instances, intraocular chemotherapy (bevacizumab) has been used. Systemic chemotherapy is the treatment of choice when synchronous metastases are present, and hormonal therapy is indicated in hormone-sensitive tumors.

Overall, treatment strategies can be tailored based on clinical presentation, extent of metastatic disease, and tumor IHC status [12].

Orbital metastases of breast cancer are characterized by their growth progression over 2 months on average [2] and the prognosis is typically poor, with reported survival times ranging from 1 to 116 months, averaging ~31 months [12].

Therefore, long-term surveillance will be required to establish effective remission and any long-term side effects.

## Conclusion

The presented case of orbital metastasis originating from ductal breast carcinoma underscores the importance of vigilance in long-term surveillance for breast cancer patients, as metastatic occurrences can manifest years post-initial diagnosis. Despite its rarity, orbital metastasis should be considered in the differential diagnosis of ocular symptoms, particularly in patients with a history of breast carcinoma. Treatment strategies primarily focus on palliation and preserving visual function, given the typically poor prognosis associated with this condition. Further research and long-term surveillance are warranted to better understand the behavior and management of orbital metastases in breast cancer patients.
